# Infectious DNAs derived from insect-specific flavivirus genomes enable identification of pre- and post-entry host restrictions in vertebrate cells

**DOI:** 10.1038/s41598-017-03120-1

**Published:** 2017-06-07

**Authors:** Thisun B. H. Piyasena, Yin X. Setoh, Jody Hobson-Peters, Natalee D. Newton, Helle Bielefeldt-Ohmann, Breeanna J. McLean, Laura J. Vet, Alexander A. Khromykh, Roy A. Hall

**Affiliations:** Australian Infectious Diseases Research Centre, School of Chemistry and Molecular Biosciences, The University of Queensland, St Lucia, 4072 Queensland Australia

**Keywords:** Restriction factors, West nile virus

## Abstract

Flaviviruses such as West Nile virus (WNV), dengue virus and Zika virus are mosquito-borne pathogens that cause significant human diseases. A novel group of insect-specific flaviviruses (ISFs), which only replicate in mosquitoes, have also been identified. However, little is known about the mechanisms of ISF host restriction. We report the generation of infectious cDNA from two Australian ISFs, Parramatta River virus (PaRV) and Palm Creek virus (PCV). Using circular polymerase extension cloning (CPEC) with a modified OpIE2 insect promoter, infectious cDNA was generated and transfected directly into mosquito cells to produce infectious virus indistinguishable from wild-type virus. When infectious PaRV cDNA under transcriptional control of a mammalian promoter was used to transfect mouse embryo fibroblasts, the virus failed to initiate replication even when cell entry steps were by-passed and the type I interferon response was lacking. We also used CPEC to generate viable chimeric viruses between PCV and WNV. Analysis of these hybrid viruses revealed that ISFs are also restricted from replication in vertebrate cells at the point of entry. The approaches described here to generate infectious ISF DNAs and chimeric viruses provide unique tools to further dissect the mechanisms of their host restriction.

## Introduction

The *Flavivirus* genus of the *Flaviviridae* family, encompasses a diverse array of viruses, which are responsible for a number of significant mosquito-transmitted diseases such as West Nile fever and encephalitis, dengue and Zika fever and Japanese encephalitis. These small enveloped viruses contain a ~11 kb positive sense, single-stranded RNA genome with a single open reading frame (ORF) flanked by 5′ and 3′ untranslated regions (UTRs). The viral ORF is translated into a single polyprotein, and post-translationally cleaved into three structural (C, prM and E) and seven non-structural proteins (NS1-NS5)^1^. Many flaviviruses are transmitted between mosquitoes and vertebrates, relying on replication in both hosts for maintaining their natural transmission cycle. However, a large group of insect-specific flaviviruses (ISFs), which replicate exclusively in mosquitoes have more recently been discovered^2–5^. These viruses appear to be transmitted vertically between mosquitoes with no requirement for a vertebrate intermediate. The advent of deep sequencing methods, sensitive reverse transcription (RT) PCR assays using flavivirus generic primers and the development of broad-spectrum diagnostic tools, such as monoclonal antibodies (mAbs) to viral dsRNA intermediates, have seen the isolation of many new ISFs from various regions around the world^3, 6–11^. These interesting viruses thus provide a unique model to investigate the molecular basis of their restriction to an insect host and efficient mode of vertical transmission. This knowledge will provide new insights into the evolution of flaviviruses. There is also the potential for ISFs to benefit public health as natural bio-control agents that suppress the transmission of vertebrate-infecting flaviviruses (VIFs) in mosquito populations^3, 4, 9, 12^.

The mechanism involved in the insect cell-restricted tropism of ISFs is currently unknown. In-depth investigation into the viral factors contributing to host-restriction of ISFs requires the generation of full-length infectious clones, which can then be readily manipulated to determine the effects of individual genes or RNA sequences on host cell permissiveness. However, this process has been traditionally encumbered by the toxicity of full-length viral cDNAs in bacteria. Various alternative approaches have been employed to overcome this problem including the use of low copy number plasmids^13, 14^, cosmid vectors^15^, *in vitro* ligation^16^, and insertion of introns^17, 18^. However, these approaches are time and labour intensive, and are prone to non-specific mutations during plasmid amplification in bacteria or *in vitro* RNA transcription. Our recently described, novel bacterium-free approach overcomes many of these pitfalls and was used to rapidly assemble flavivirus infectious cDNAs for the Kunjin strain of West Nile virus (WNV_KUN_)^19, 20^. Circular Polymerase Extension Cloning (CPEC) operates without the need for restriction enzyme digestion, ligation, or single-stranded homologous recombination^21^. Our most recent iteration of the CPEC system includes the removal of all bacterial regulatory sequences from the CPEC linker fragment which circularises the flavivirus genome via the two UTR’s, and contains a CMV promoter to drive transcription of the viral RNA^19^. This system was used to effectively generate WNV chimeric viruses, whereby the systematic and precise exchange of genes between differing strains of WNV was performed to identify the role of non-structural proteins in WNV virulence^19^. This study highlighted the efficacy of CPEC as a fast and reliable method for manipulating full-length flavivirus infectious DNA.

In its current format, the CPEC system is unsuitable for the preparation of ISF infectious cDNA due to the inability of the CMV promoter to drive the initial transcription of the viral RNA in insect cells. Here we report the generation of ISF infectious cDNAs, which produce full-length viral RNA genomes from a modified *Orgyia pseudotsugata* multicapsid nucleopolyhedrosis virus immediate-early 2 (OpIE2) promoter. We used Parramatta River virus (PaRV)^22^ and Palm Creek virus (PCV)^3^, novel Australian ISF species isolated in our lab from *Aedes vigilax* and *Coquillettidia xanthogaster* mosquitoes, respectively. The generation of PaRV and PCV CPEC constructs and the successful recovery of corresponding infectious viruses in insect cells represent major steps forward in ISF research and provides unique tools to identify the stages of vertebrate cell infection where ISF host-restriction takes place. We also report the use of the CPEC approach to generate chimeric viruses between ISFs and VIFs, thus further expanding the tools to investigate mechanisms of ISF restriction as well as providing potential platform for generating recombinant viruses between ISFs and VIFs as candidates for safe vaccines and diagnostic antigens for flaviviral diseases.

## Results

### CPEC insect promoter optimisation

**Figure 1:**
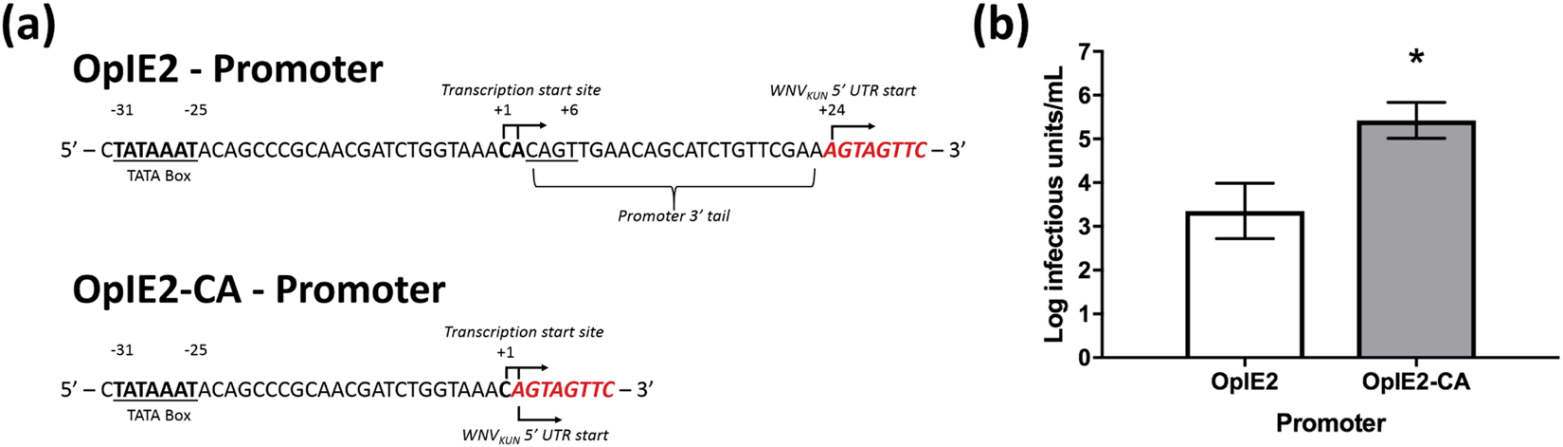
OpIE2 promoter optimisation. (**a**) Schematic of the 3′ termini of the OpIE2 and OpIE2-CA promoters. (**b**) TCID_50_ of P_0_ supernatants from C6/36 cells transfected with WNV_KUN_ CPEC containing either the OpIE2 or OpIE2-CA promoter (n = 3 biological replicates for each construct). Supernatants harvested 5 days post-transfection indicate that the OpIE2-CA promoter yields approximately 100-fold higher titres than the OpIE2 promoter. Error bars represent standard deviation and asterisks indicate significance (P value < 0.05; two-tailed t-test).

Due to the restriction of ISF replication to insect cells, a modification of the existing CPEC protocol, which employs a CMV promoter for mammalian systems, was required. The OpIE2 promoter originally described by Blissard and Rohrmann^23^ was chosen to drive transcription of CPEC-assembled infectious cDNA in insect cells. Previous comparative analyses using this promoter have shown it to be highly active in multiple insect cell lines^24, 25^. To convert the recently described CPEC system for the generation of infectious flavivirus DNA in vertebrate cells^19^ to a system for use in mosquito cells, the UTR-linker fragment was modified to replace the existing CMV promoter with the complete OpIE2 promoter. A truncated version of this promoter, lacking the 23 nucleotides comprising the promoter 3′ tail downstream of the transcription start site (OpIE2-CA), was also designed to reduce the number of extra nucleotides added to the 5′ end of the transcribed flavivirus genome (Fig. 1a). The modified OpIE2 UTR-linkers were assembled by CPEC with cDNA fragments from WNV_KUN_ and directly transfected into C6/36 cells. A TCID_50_ of recovered infectious virus following transfection of WNV_KUN_ CPEC constructs into C6/36 cells indicated that passage 0 (P_0_) titres were approximately 100-fold higher when using the truncated promoter (OpIE2-CA - 10^5.42^ IU/mL) compared to the full-length version (OpIE2 - 10^3.35^ IU/mL) (Fig. 1b).

### Generation of PaRV infectious DNA construct using circular polymerase extension cloning

**Figure 2:**
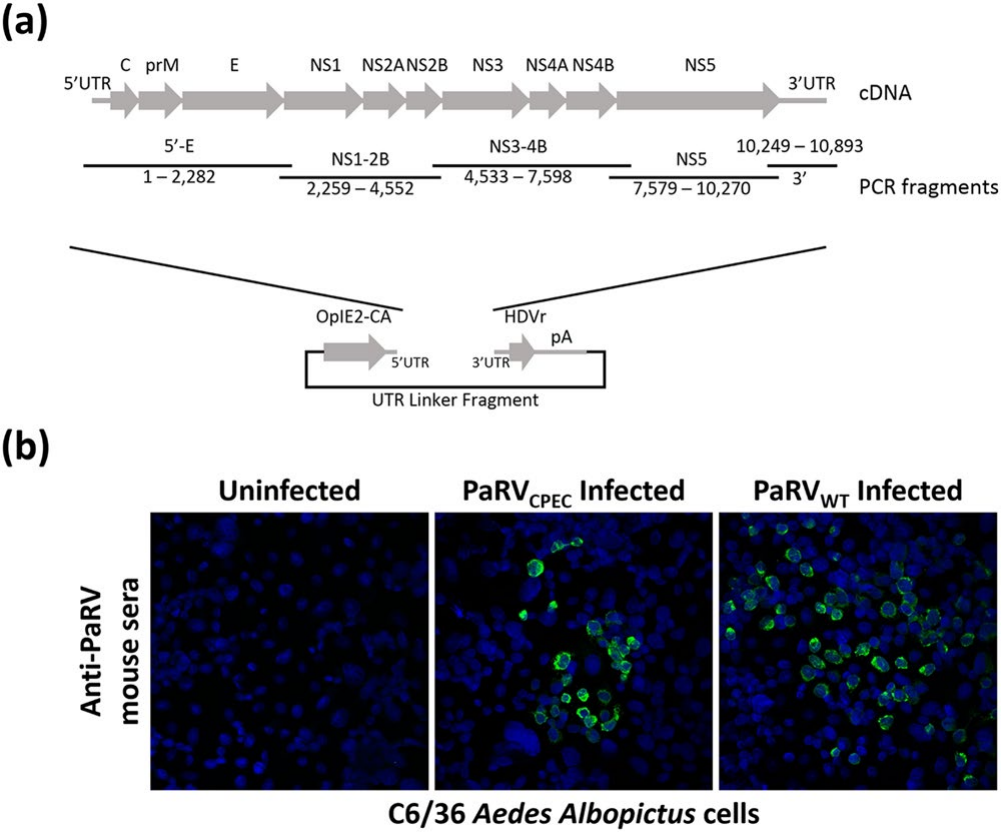
Generation of PaRV using CPEC. (**a**) A schematic representation for the assembly of infectious DNA for PaRV by CPEC reaction. (**b**) Visualisation of PaRV replication in mosquito (C6/36) cell monolayers inoculated with either a MOI of 0.1 PaRV_WT_ or undiluted P_0_ PaRV_CPEC_. Monolayers were fixed 72 hrs post-infection. IFA analysis was performed by probing with PaRV mouse anti-sera. The nucleus of each cell was stained with Hoechst 33342. Images were taken at ×40 magnification.

The modified OpIE2-CA UTR-linker fragment was selected for generating the PaRV infectious cDNA by CPEC (Fig. 2a). Primers used for constructing the PaRV cDNA library were designed to anneal at the junctions between viral genes predicted from the PaRV genome sequence^22^ (Fig. 2a). CPEC-derived PaRV (PaRV_CPEC_) was successfully recovered from two independent transfections of C6/36 cell cultures, and the identity of the progeny virus confirmed by RT-PCR and Sanger sequencing of approximately 1.5 kb of the C-prM-E region of the viral genome. Supernatant from the PaRV_CPEC_-transfected culture (P0) was also inoculated on to fresh C6/36 cells, and PaRV-specific antigens detected at 3 days post-infection by IFA using anti-PaRV mouse serum^22^ to demonstrate successful replication of the progeny PaRV_CPEC_ virus (Fig. 2b).

### CPEC-derived PaRV is phenotypically identical to wild-type PaRV

**Figure 3:**
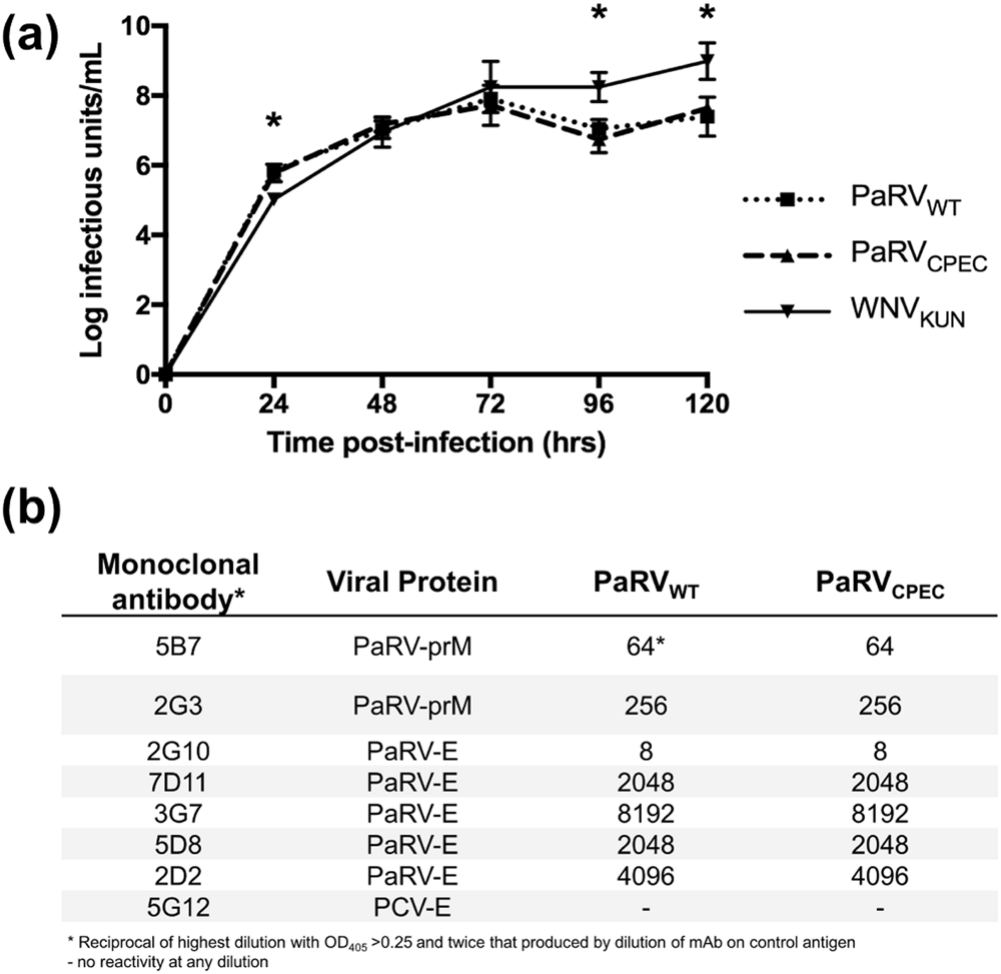
Phenotypic analysis of PaRV_WT_ and PaRV_CPEC_. (**a**) Comparative growth kinetics of PaRV_WT_, PaRV_CPEC_ and WNV_KUN_ in C6/36 cells. C6/36 cells were infected with either PaRV_WT_, PaRV_CPEC_ or WNV_KUN_ at a MOI of 0.1. Infectious titres at each time point were determined by titration of culture supernatant on to fresh C6/36 cells with infection detected using fixed cell ELISA. Error bars represent standard deviation and asterisks indicate significance (P value < 0.001) as determined by a two-way ANOVA. (**b**) Comparison of reciprocal titres for the reactivity of a panel of anti-PaRV (5B7, 2G3, 2G10, 7D11, 3G7, 5D8, 2D2 and 1E5) and anti-PCV control (5G12) mAbs to PaRV_WT_ and PaRV_CPEC_.

PaRV_CPEC_ was further assessed by two methods to confirm that it was phenotypically identical to PaRV_WT_. In a growth kinetics assay, PaRV_CPEC_ displayed a growth profile identical to PaRV_WT_, with no significant difference in the titres at any time point (two-way ANOVA). Both viruses replicated rapidly in the first 24 hrs (PaRV_CPEC_ 10^5.78^ IU/mL; PaRV_WT_ 10^5.86^ IU/mL), reaching a peak titre at 72 hrs (PaRV_CPEC_ 10^7.72^ IU/mL; PaRV_WT_ 10^7.91^ IU/mL), after which the virus titre plateaued (Fig. 3a) similar to our previously reported results for wild-type PaRV^22^. The titre of both PaRV viruses at 24 hours was significantly higher when compared to WNV_KUN_, however, by four days post-infection WNV_KUN_ (10^8.24^ IU/mL) produced a significantly (two-way ANOVA; P < 0.001) higher titre (PaRV_WT_ -10^7.39^ IU/mL; PaRV_CPEC_ - 10^7.63^ IU/mL). This was also consistent with our previous findings^22^. Further antigenic analysis using a panel of monoclonal antibodies (mAb) that were generated to native PaRV prM and E proteins, confirmed that each epitope was conserved between PaRV_WT_ and PaRV_CPEC_ (Fig. 3b). Comparison of levels of infectious virus derived from P_0_ cultures transfected with either purified PaRV RNA or a PaRV CPEC reaction also revealed comparable titres (10^7.80^ IU/mL and 10^6.97^ IU/mL, respectively) after a 5 day incubation.

### Using CPEC to bypass viral entry and IFN response-deficient cells to remove restriction by the innate immune response did not permit PARV replication in vertebrate cells

**Figure 4:**
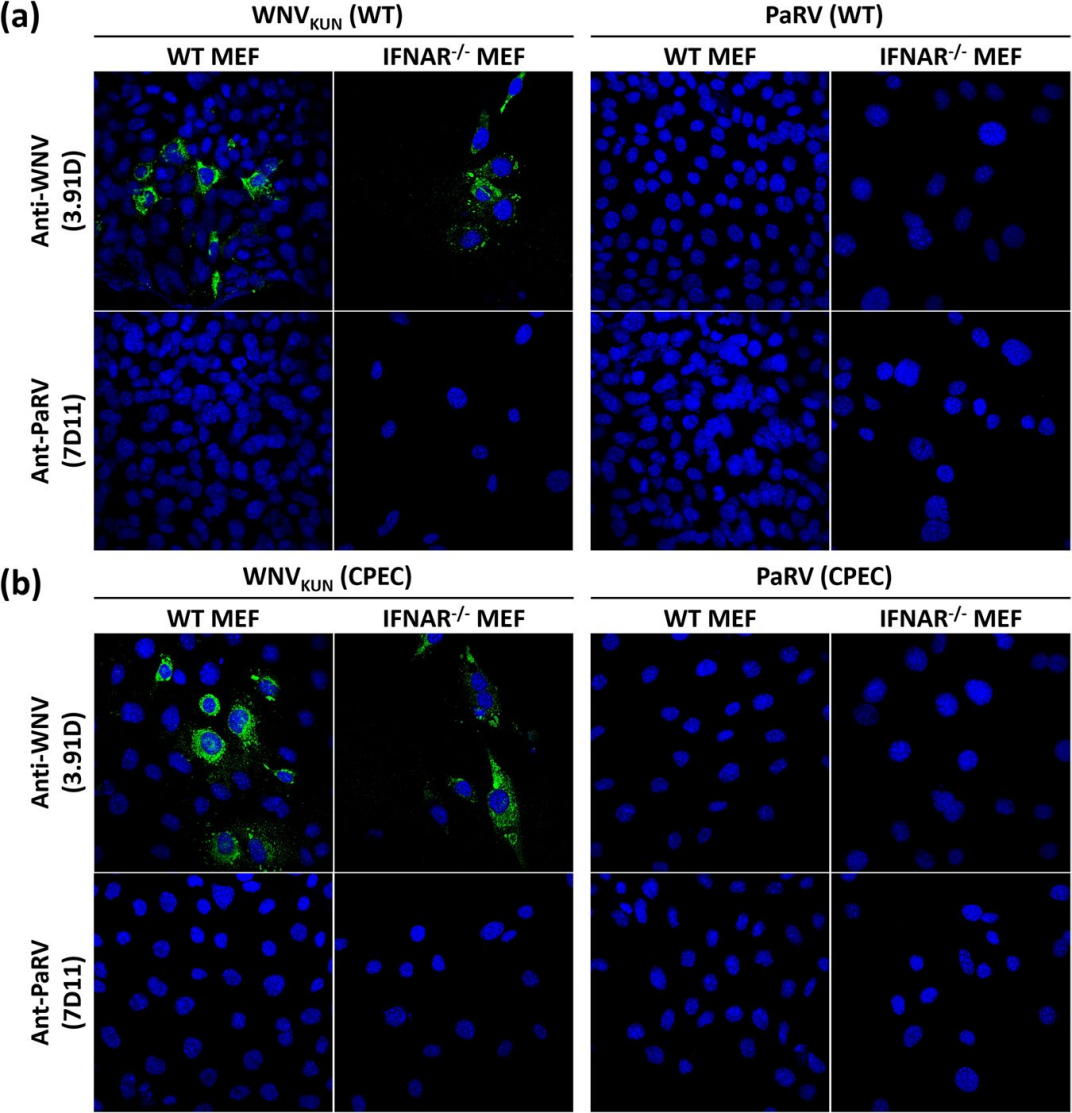
Viral replication analyses in WT and IFNAR^−/−^ MEF cells. (**a**) Cells infected at an MOI of 1 or transfected CPEC constructs of either WNV_KUN_ or PaRV with a CMV promoter. (**b**) Cells transfected with genomic RNA of either PaRV, WNV_KUN_, PCV, PCV/WNV_KUN_ -prME or WNV_KUN_/PCV-prME. Monolayers were fixed 72 hrs post-infection. IFA analysis was performed by probing with anti-PaRV (7D11) and anti-WNV E (3.91D) mouse monoclonal antibodies. The nucleus of each cell was stained with Hoechst 33342. Images were taken at ×40 magnification.

Barriers to ISF infection and replication in vertebrate cells may occur at any stage of virus entry, replication and release or could be due to restriction by the host innate immune response^5, 26^. To examine the potential roles for viral entry and innate immune response in restriction of ISFs in vertebrate cells, wild-type (WT) and IFN-α/β receptor knockout (IFNAR^−/−^) mouse embryonic fibroblasts (MEFs), were transfected with PaRV CPEC DNA driven by a CMV promoter or a similarly prepared WNV_KUN_ CPEC DNA. Infection with PARV and WNV_KUN_ virus was also employed as a control. IFA analysis of both virus-infected and CPEC-transfected cells revealed no observable replication of PaRV in either WT or IFNAR^−/−^ MEFs (Fig. 4). In contrast, both cell lines displayed WNV_KUN_ replication whether infected with virus or transfected with CPEC DNA. To confirm the lack of initiation of PaRV replication in vertebrate cells, purified PaRV virion RNA was also used to transfect WT MEFs, IFNAR^−/−^ MEFs, and BHK cells. IFA analysis of the transfected cells revealed no PaRV replication in any of the vertebrate cell lines while GFP expression was clearly observed in BHK cells transfected with a control WNV_KUN_ replicon RNA encoding GFP (See Supplementary Fig. S1). In contrast, C6/36 cells transfected with PaRV RNA showed clear viral replication. These data demonstrate that PaRV fails to initiate replication even after bypassing viral entry and removing the type I IFN response.

### ISF/VIF chimeric viruses as a tool to study viral host restriction

The generation of chimeric viruses between ISFs and VIFs would provide useful tools to elucidate the molecular basis of ISF host-restriction. However, repeated efforts using CPEC to generate chimeric viral genomes containing either PaRV prME genes on a WNV_KUN_ genomic backbone (WNV_KUN_/PaRV-prME) or WNV_KUN_ -prME genes on a PaRV genomic backbone (PaRV/WNV_KUN_-prME) were unsuccessful. Nevertheless, we were able to generate a mutant PaRV virus with an in-frame, 15 nucleotide stretch of the prM gene replaced with the corresponding sequence of WNV_KUN_ (PaRV_CPECM1_). The progeny virus was confirmed by sequencing of the prM gene (See Supplementary Table S1), and although PaRV_CPECM1_ contained an additional codon, the virus grew to similar titres to PaRV_CPEC_ (10^6.3^ IU/mL) demonstrating that CPEC could be used to produce infectious, recombinant PaRV.

**Figure 5:**
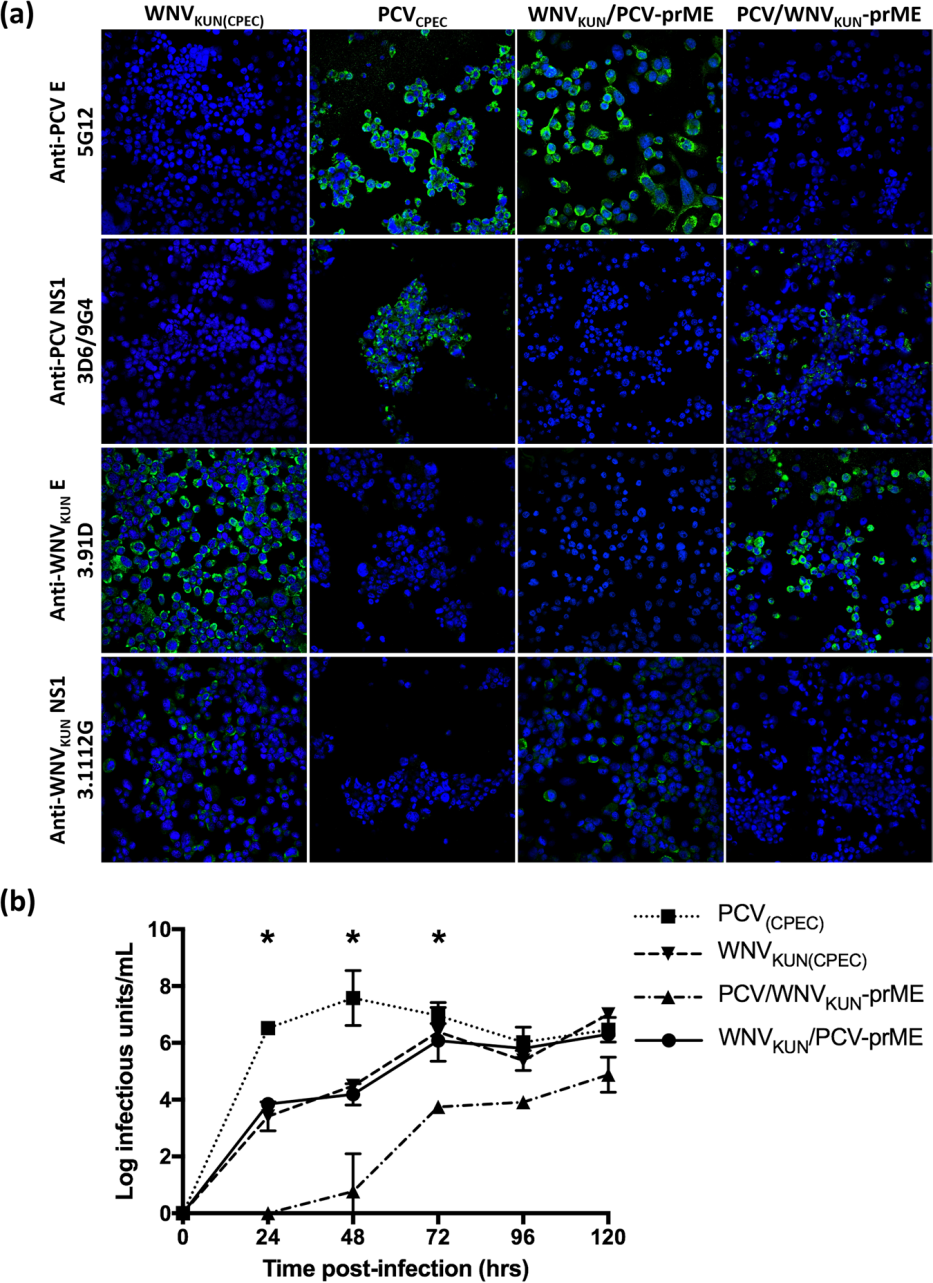
Generation and characterisation of chimeric viruses between ISFs and VIFs. (**a**) Visualisation of C6/36 cells transfected with CPEC constructs of either WNV_KUN_, PCV, WNV_KUN_/PCV-prME or PCV/WNV_KUN_-prME chimeric viruses. IFA analysis was performed by probing with anti-PCV E (5G12), anti-PCV NS1 (3D6/9G4), anti-WNV_KUN_ E (3.91D) and anti-WNV_KUN_ NS1 (3.1112G) mouse monoclonal antibodies. Monolayers were fixed 5 days post-transfection. The nucleus of each cell was stained with Hoechst 33342. Images were taken at ×40 magnification. (**b**) Comparative growth kinetics of WNV_KUN(CPEC)_, PCV_CPEC_, WNV_KUN_/PCV-prME and PCV/WNV_KUN_-prME in C6/36 cells. C6/36 cells were infected with either virus at a MOI of 0.1. Infectious titres at each time point were determined by titration of culture supernatant on fresh C6/36 cells with infection detected using fixed cell ELISA. Error bars represent standard deviation and asterisks indicate significance (P value < 0.001) as determined by a two-way ANOVA.

To assess whether the incompatibility between PaRV and WNV was a trait shared by other ISFs, additional constructs were prepared. Using the same strategy employed for construction of PaRV_CPEC_, an infectious DNA construct of PCV was also successfully generated by CPEC and characterised (Fig. 5). Similarly, chimeric cDNAs containing the PCV prME genes on a WNV_KUN_ genomic backbone (WNV_KUN_/PCV-prME) and WNV_KUN_ -prME genes on a PCV genomic backbone (PCV/WNV_KUN_-prME) were produced by CPEC. In contrast to the non-viable PaRV-WNV chimeric constructs, WNV_KUN_/PCV-prME and PCV/WNV_KUN_-prME chimeric cDNAs generated viable viruses in CPEC DNA-transfcted C6/36 cells. IFA analysis of WNV_KUN_/PCV-prME virus revealed expression of both PCV E and WNV_KUN_ NS1 protein, but not PCV NS1 or WNV_KUN_ E proteins (Fig. 5a). The inverse was observed for the PCV/WNV_KUN_-prME virus indicating that the correct chimeric viruses were produced. The authenticity of the PCV_CPEC_, WNV_KUN_/PCV-prME and PCV/WNV_KUN_-prME chimeric viruses were further verified as described earlier by Sanger sequencing of a 1 kb C-prME region of the viral RNA isolated from passage P_1_ C6/36 supernatant.

Growth kinetics assays indicated PCV_CPEC_ replicated rapidly in the first 24 hrs (PCV_CPEC_ 10^6.52^ IU/mL), reaching significantly (two-way ANOVA; P < 0.001) higher tires than WNV_KUN(CPEC)_, WNV_KUN_/PCV-prME and PCV/WNV_KUN_-prME. The titre peaked at 48 hrs (PCV_CPEC_ 10^7.58^ IU/mL), after which the virus titre plateaued (Fig. 5b) consistent with our previous results with wild-type PCV^12^. WNV_KUN_/PCV-prME displayed a growth profile identical to WNV_KUN_, with no significant difference in the titres at any time point. Both WNV_KUN_ and WNV_KUN_/PCV-prME peaked 72 hrs post infection (WNV_KUN(CPEC)_ 10^6.39^ IU/mL, WNV_KUN_/PCV-prME 10^6.08^ IU/mL). The titres for PCV/WNV_KUN_-prME were significantly lower than the other viruses tested throughout the study, reaching peak titre 72 hrs post infection (PCV/WNV_KUN_-prME 10^3.74^ IU/mL). However, in cultures incubated for 7 days the virus reached a titre of 10^6.3^ IU/mL.

**Figure 6:**
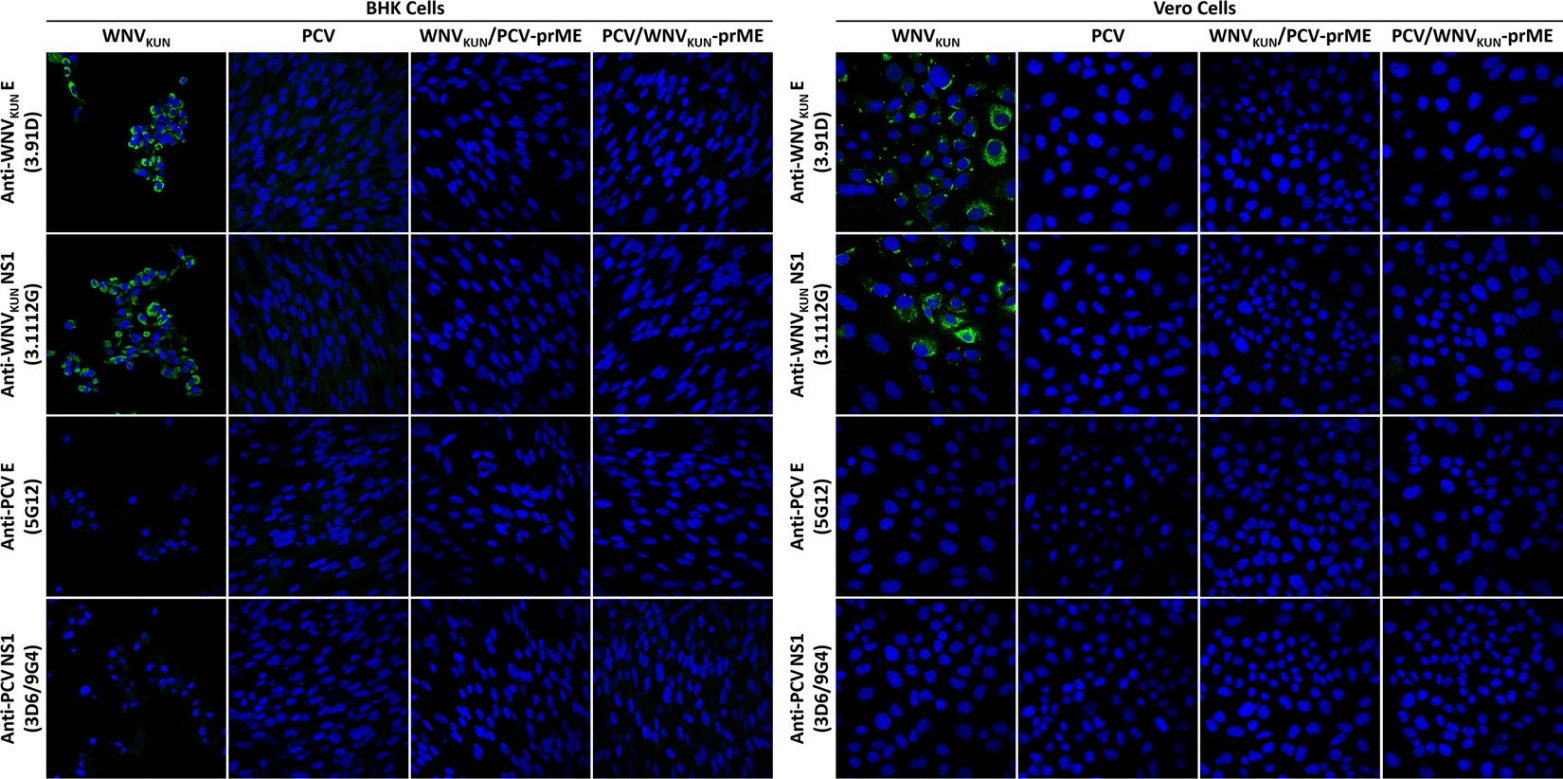
Visualisation of BHK and Vero cells infected with either WNV_KUN_, PCV, PCV/WNV_KUN_-prME or WNV_KUN_/PCV-prME at an MOI of 1. Monolayers were fixed 72 hrs post-infection. IFA analysis was performed by probing with anti-PCV E (5G12), anti-PCV NS1 (3D6/9G4), anti-WNV E (3.91D) and anti-WNV NS1 (3.1112G) mouse monoclonal antibodies. The nucleus of each cell was stained with Hoechst 33342. Images were taken at ×40 magnification.

To further investigate the role of viral entry in ISF host restriction, BHK, Vero and C6/36 cells were infected with MOI = 1 of P_1_ WNV_KUN(CPEC)_, PCV_CPEC_, WNV_KUN_/PCV-prME or PCV/WNV_KUN_-prME. IFA analyses of cells revealed that while all four viruses readily replicated in C6/36 cells (See Supplementary Fig. S2), only WNV_KUN_ exhibited productive infection in BHK and Vero cells (Fig. 6). Despite containing the replicative proteins of WNV_KUN_, WNV_KUN_/PCV-prME failed to replicate in vertebrate cells. Similarly, PCV/WNV_KUN_-prME was also unable to replicate in vertebrate cells despite containing WNV_KUN_ structural proteins, which would allow the virus entry into the cells. Similar results were also observed when WT and IFNAR^−/−^ MEF cells were infected with PCV_CPEC_, WNV_KUN_/PCV-prME or PCV/WNV_KUN_-prME at an MOI of 10 (*Data not shown*). These results suggest that a restriction barrier for PCV likely occurs at both the point of cell entry and at the RNA replication stage.

## Discussion

Here we report an infectious ISF cDNA constructed using a modification of our previously described CPEC method^19, 20^. Infectious PaRV virions were successfully recovered from C6/36 cells transfected with a CPEC-assembled PaRV infectious cDNA containing a modified OpIE2 promoter. The recovered CPEC-derived PaRV was phenotypically identical to the wild-type virus. While attempts to produce chimeric viruses between PaRV and WNV_KUN_ were unsuccessful, demonstration that viable ISF/VIF chimeric viruses could be generated was provided through the successful production of chimeric WNV_KUN_/PCV-prME and PCV/WNV_KUN_-prME viruses. Thus, we provide a proof-of-concept that viable ISF/VIFs can be successfully generated. Furthermore, the methods and reagents described here provide a useful set of tools to enable the investigation of viral determinants of host-restriction in ISFs.

Despite the power of reverse genetics as a tool for understanding the aspects of viral replication and pathogenesis, as well as vaccine development, few infectious clones of insect-specific viruses have been reported. The first publication describing an insect-specific virus infectious clone was for a full-length cDNA clone of Culex flavivirus (CxFV)^27^. Additionally, infectious clones of Eilat virus (EILV), a unique insect-specific alphavirus, were transfected into vertebrate cells to elucidate that its host-restriction was present even as early as RNA replication^28, 29^. The EILV infectious clones were also chimerised with Sindbis virus (SINV) and chikungunya virus (CHIKV) structural genes to determine that EILV was restricted both at entry and genomic RNA replication levels in vertebrate cells and for developing novel and safe means of generating diagnostic antigen and vaccines for CHIKV^30–32^. More recently, an infectious clone of Niénokoué virus (NIEV), a flavivirus isolated from mosquitoes sampled in Côte d’Ivoire, and a chimeric virus containing the NIEV structural genes on a yellow fever virus (YFV) backbone were used to identify the stages of ISF restriction in vertebrate cells^33^. Infectious clones of two mosquito-specific negeviruses have also been reported^34, 35^. In each of these examples, infectious clones and chimeric constructs (for EILV) were generated using standard cDNA subcloning techniques into suitable plasmid(s) along with the incorporation of an SP6 or T7 RNA polymerase promoter, followed by *in vitro* transcription of the RNA transcript and subsequent transfection of the transcript into cells^36, 37^.

We believe that the generation of an infectious ISF cDNA construct using CPEC, as described herein, represents a significant improvement in the methodologies for generating recombinant ISFs. Our inclusion of the OpIE2 insect promoter to drive the transcription of the PaRV genome negated the requirement for *in vitro* transcription. However, initial transfections of PaRV_CPEC_ containing the full-length promoter sequence yielded significantly (P value < 0.05) lower titres compared to PaRV_WT_, suggesting that the OpIE2 promoter required optimisation in the context of driving transcription of the PaRV genome. Indeed, the 5′ and 3′ UTRs, which flank the coding regions of the flaviviral genome, are a prerequisite for initiating RNA replication^38^. Both UTRs contain highly-defined stem-loop structures which have been shown to interact with viral replicative proteins such as NS5 to initiate RNA transcription^39–41^. Importantly, the functionality of the 5′ UTR is likely to be dependent on the specificity of the upstream promoter to initiate transcription from the first nucleotide of the viral genome. Thus, it is likely the extra nucleotides added to the 5′ end of viral RNA following transcription via the full length OpIE2 promoter was rendering the virus less replication competent. Previous deletion analyses of the OpIE2 promoter had shown that only nucleotides up to −275 from the transcription start site were necessary for promoter function^42^. The truncation of 23 nucleotides from the 3′ end in the modified promoter (OpIE2-CA), between the transcription start site and 5′ terminal nucleotide of the viral genome sequence, ensured an authentic 5′ UTR sequence of the PaRV RNA with no additional bases. OpIE2 promoter transcription has been shown to initiate equally from either the +1 or +2 transcription start nucleotides resulting in some transcripts containing a single additional nucleotide at the 5′ terminus. However, previous studies have shown that a single additional nucleotide at the distal end of the flavivirus 5′ UTR is lost early during viral replication and had no significant side effects^43^. Thus, the increase in viral titre following the optimisation of the OpIE2 promoter was most likely due to the deletion of the promoter 3′ tail region immediately downstream of the transcription start site resulting an authentic 5′ UTR sequence in the transcribed PaRV genome. The availability of CPEC to easily generate infectious DNAs of ISFs under transcription control of both insect and mammalian promoters also provides a powerful tool to investigate the mechanisms of ISF host restriction. Thus, transfection of IFNAR^−/−^ MEFs with the PaRV CPEC construct incorporating a CMV promoter allowed us to assess viral replication efficiency without the requirement of the virus to enter cells and in the absence of downstream JAK-STAT signalling pathways and induction of IFN-stimulated genes (ISGs)^44^.

To create tools for the identification of the viral factors associated with the mechanisms underlying host-restriction in ISFs, chimeric CPEC constructs of PaRV and WNV_KUN_ with swapped prM-E genes were designed. The capsid gene was not included in the exchanged structural genes, as it has been shown to contain key elements such as cyclisation sequences, which are critical for the appropriate folding and configuration of flaviviral UTRs during replication^45, 46^. Our failure to generate infectious PaRV/WNV_KUN_ chimeras, despite repeated attempts and serial passaging of the transfected cultures, may be due to a lack of recognition of key cleavage motifs at the junctions between the PaRV and WNV_KUN_ genome components (i.e. C-prM and E-NS1) by host signalases. However, *in silico* analyses of the proposed cleavage sites in the deduced polyprotein sequence of the non-viable chimeric viral genomes revealed that the predicted cleavage efficiency by signalase was similar to that of the corresponding sites in the wild-type parental viruses (*Data not shown*). Interestingly the recovery of viable virus from PaRV_CPECM1_, which contains a shorter substitution from WNV_KUN_ suggested that chimerisation between the two viruses is also possible on a much smaller scale.

Our success in generating an infectious chimeric virus containing genes from WNV_KUN_ on a PCV genetic backbone is the first report of a viable ISF chimera expressing VIF structural genes. Previous attempts to generate chimeric viruses using ISFs and VIFs have proved difficult^47^, with only recent developments leading to the generation of a chimeric VIF with ISF structural genes^33^. Our demonstration that the WNV_KUN_/PCV-prME chimera could not infect vertebrate cells despite containing WNV_KUN_ replicative genes and UTRs, indicated that the ISF structural proteins were associated with host restriction at the stage of cell entry. Indeed, the structure of the receptor-binding domain III of the E protein of most ISFs is radically altered compared to that of VIFs, suggesting it may be associated with binding to mosquito-specific cell receptors^22, 26^. In contrast, the failure of the PCV/WNV_KUN_-prME chimera to initiate replication in vertebrate cells, despite possessing the structural proteins of WNV_KUN_, indicated that additional barriers to ISF replication exist in vertebrate cells, such as IFN-independent antiviral responses and/or incompatible virus-host cell interactions that are required for virus replication^48^. These findings are consistent with a recent report showing an absence of viral replication in IFNAR^−/−^ MEFs inoculated with the ISF Kamiti River virus (KRV). However, that study did show trace levels of virus replication in IRF 3, 5, 7^−/−^ MEFs, further suggesting a role for IFN-independent innate responses in restricting KRV replication^48, 49^. A more recent study using a NIEV reporter replicon and a YFV/NIEV chimeric virus also showed a lack of viral replication in vertebrate cells inoculated with the YFV/NIEV chimeric virus or transfected with NIEV replicon RNA^33^. However, limited replication with a lack of infectious particle production was observed in cells transfected with YFV/NIEV RNA. The authors also concluded that ISFs were unable to enter vertebrate cells and that an additional intracellular barrier to replication was likely associated with a lack of interaction between ISFs and vertebrate host cell factors.

In this study the development of a CPEC system to generate infectious ISF DNAs and chimeric viruses, not only allowed the identification of multiple stages of ISF growth restriction in vertebrate cells, but also provides an approach to rapidly prepare infectious cDNA constructs for a variety of ISFs and other insect-specific viruses. This will allow the fast and efficient production of mutant and chimeric viruses to further dissect the molecular mechanisms of host restriction in ISFs. Our generation of a panel of mAbs reactive to ISF viral proteins further provides a complimentary set of reagents to facilitate these studies.

## Materials and Methods

### Cell Culture

C6/36 (*Aedes albopictus*) cells were cultured at 28 °C in RPMI 1640 medium supplemented with 5% foetal bovine serum (FBS). Wild-type (WT) and interferon-α/β receptor deficient (IFNAR^−/−^) mouse embryonic fibroblasts (MEF), baby hamster kidney (BHK) and African green monkey (Vero) cells were cultured in Dulbecco’s modified Eagle’s medium (DMEM) supplemented with 5% FBS and grown at 37 °C with 5% CO_2_. All media contained 50 U penicillin/mL, 50 mg streptomycin/mL and 2 mM L-glutamine.

### Virus Culture

Parramatta River virus (PaRV - NC_027817.1), Palm Creek virus (PCV - KC505248.1) and West Nile virus strain Kunjin (WNV_KUN_ - AY274504) viral stocks were propagated in C6/36 cells incubated at 28 °C for 5–7 days. Viral titres were assessed by infection of C6/36 cells with 10-fold serial dilutions of supernatant in 96-well plates and incubation for 5 days. The cell supernatant was aspirated and the monolayers fixed with acetone (20% acetone, 0.02% bovine serum albumin (BSA) in phosphate buffered saline (PBS). PaRV was detected by enzyme-linked immunosorbent assay (ELISA) using the PaRV-specific monoclonal antibody (mAb) 7D11, PCV using PCV-specific mAb 5G12 and WNV_KUN_ using mAb 4G2^50^. Virus titres were calculated as 50% tissue culture infective dose (TCID_50_) using the methods described by Reed and Muench^51^.

### Preparation of monoclonal antibodies to PaRV and PCV

All animal experiments were conducted according to the guidelines set out in the Australian Code for the Care and Use of Animals for Scientific Purposes 8th edition (2013) and were approved by The University of Queensland Animal Ethics Committee - approval #SCMB/329/15/ARC. All animal procedures where necessary were performed under ketamine:xylazil anaesthesia. Six-week old BALB/c mice (Animal Resources Centre, Murdoch, Western Australia, Australia) were immunised twice via the subcutaneous route with purified PaRV or PCV virions, along with the inulin-based adjuvant Advax (Vaxine Ltd, Adelaide, Australia). Mice were kept on clean bedding and given food and water *ad libitum*. The mice were boosted with PaRV or PCV virions by intravenous injection four days prior to harvesting of the spleen. Fusion of the spleen cells with NS0 myeloma cells (European Collection of Cell Cultures) was performed as previously described^52^. Hybridomas secreting antibodies reactive to PaRV- or PCV-infected C6/36 cells were identified by ELISA using previously described methods^53^. The target protein of each mAb was determined using PaRV- or PCV-infected cell lysates in Western blot using previously published methods^53^. Reaction of selected monoclonal antibodies with antigens of wild-type and CPEC derived-PaRV were analysed by fixed cell ELISA^53^.

### Generation and characterisation of a modified OpIE2 insect promoter

OpIE2 promoter sequences characterised by Blissard and Rohrmann^23^ were synthesised as gBlocks Gene Fragments (*IDT*). These fragments were cloned into a previously generated plasmid containing a sequence which, when expressed by itself, linked the UTR regions of the viral genome together^19^. A Gibson Assembly Master Mix (*NEB*) was used. Constructs were transformed into DH5α competent *E*. *coli*, and colony PCR using Taq DNA Polymerase (*NEB*) conducted to screen for viable colonies. Plasmids were extracted from overnight cultures of positive colonies using a NucleoSpin Plasmid Miniprep kit (*Macherey*-*Nagel*). Extracted plasmids were sequenced by the Australian Genome Research Centre.

### Generation of viruses by CPEC

CPEC constructs were generated based on previously described methods^19^. Briefly, viral RNA was extracted using a NucleoSpin RNA Virus kit (*Macherey*-*Nagel*) and converted to cDNA using a qScript cDNA SuperMix (*Quantabio*). For each CPEC assembly, 0.1 pmol of each viral cDNA fragment was added to a Q5 PCR reaction (*NEB*) as per the manufacturer’s instructions. Primers used are available upon request. Thermal cycling was carried out at 98 °C for 2 mins (one cycle), 98 °C for 30 secs, 55 °C for 30 secs, 72 °C for 6 mins (2 cycles), 98 °C for 30 secs, 55 °C for 30 secs, 72 °C for 8 mins (ten cycles). The entire CPEC reaction was transfected into cells and the passage 0 (P_0_) cell culture supernatants harvested and stored at −80 °C, five days post-transfection. Additionally, cDNA of any progeny virus was generated as before and amplified with Q5 High-Fidelity DNA Polymerase (*NEB*) prior to sequencing by the Australian Genome Research Centre.

### Growth Kinetics

C6/36 cells seeded at a density of 1 × 10^5^ were inoculated in triplicate with a P_1_ CPEC-derived and P7 wild-type virus stock at a multiplicity of infection (MOI) of 0.1. After incubation at 28 °C for 1 hr the inoculum was removed and the monolayer washed three times with sterile PBS before re-incubating at 28 °C with fresh RPMI 1640 with 2% FBS. Supernatant was harvested at 2, 24, 48, 72, 96 and 120 hrs post infection. Viral titres from each time point were determined using a TCID_50_ assay as previously described. A two way-ANOVA was performed on the results using Graphpad Prism.

### IFA

Cells seeded at a density of 1 × 10^5^ on glass coverslips in a 24-well plate were transfected or infected as required. Following a 72 hr incubation, the coverslips were fixed with ice cold 100% acetone and air dried before storing at −20 °C. Prior to staining, coverslips were blocked with blocking buffer (0.05 M Tris/HCl (pH 8.0), 1 mM EDTA, 0.15 M NaCl, 0.05% (v/v) Tween-20, 0.2% w/v casein) for 1 hr at room temperature. Coverslips were then incubated for 1 hr with primary antibody in blocking buffer. The mAbs used for this work included anti-WNV E (3.91D^54^ or 4G2^55^), anti-WNV NS1 (3.1112 G^54^ or 4G4), anti-PaRV E (7D11), anti-PCV E (5G12) and anti-PCV NS1 (3D6/9G4)^3^. Following 3 washes with PBS containing 0.05% Tween-20 (PBST), antibody binding was detected by incubation for 1 hour with Alexafluor 488-conjugated goat anti-mouse IgG (H + L) (*Invitrogen*) diluted 1:1000 in blocking buffer. A Hoechst 33342 nuclear stain (*Invitrogen*) was applied at 1:1000 for 5 mins at room temperature. Following a final 3 washes with PBST, the coverslips were mounted onto glass microscope slides using ProLong Gold Anti-fade (*Invitrogen*). All coverslips were viewed under the ZEISS LSM 510 META confocal microscope.

### Cell Transfection

C6/36 cells were transfected with DNA using Effectene (*Qiagen*) or RNA using TransMessenger (*Qiagen*), as per the manufacturer’s instructions. Wild-type and IFNAR^−/−^ MEF cells were transfected using Lipofectamine 2000 (*Invitrogen*), as per the manufacturer’s instructions. RNA from a GFP-nanoLuc fusion protein-expressing WNV replicon generated using methods previously described by Khromykh and Westaway^56^, was used as a transfection control for vertebrate cells.

## Electronic supplementary material

Supplementary Data
