# Author Correction: Infectious DNAs derived from insect-specific flavivirus genomes enable identification of pre- and post-entry host restrictions in vertebrate cells

**DOI:** 10.1038/s41598-019-44657-7

**Published:** 2019-06-05

**Authors:** Thisun B. H. Piyasena, Yin X. Setoh, Jody Hobson-Peters, Natalee D. Newton, Helle Bielefeldt-Ohmann, Breeanna J. McLean, Laura J. Vet, Alexander A. Khromykh, Roy A. Hall

**Affiliations:** Australian Infectious Diseases Research Centre, School of Chemistry and Molecular Biosciences, The University of Queensland, St Lucia, 4072 Queensland Australia

Correction to: *Scientific Reports* 10.1038/s41598-017-03120-1, published online 07 June 2017

In this Article, the legend of Figure 4 is incorrect:

“Viral replication analyses in WT and IFNAR^−/−^ MEF cells. (a) Cells infected at an MOI of 1 or transfected CPEC constructs of either WNV_KUN_ or PaRV with a CMV promoter. (b) Cells transfected with genomic RNA of either PaRV, WNV_KUN_, PCV, PCV/WNV_KUN_ -prME or WNV_KUN_/PCV-prME. Monolayers were fixed 72 hrs post-infection. IFA analysis was performed by probing with anti-PaRV (7D11) and anti-WNV E (3.91D) mouse monoclonal antibodies. The nucleus of each cell was stained with Hoechst 33342. Images were taken at ×40 magnification”.

should read:

“Viral replication analyses in WT and IFNAR^−/−^ MEF cells. (a) Cells infected at an MOI of 1 with either WT WNV_KUN_ or WT PaRV. (b) Cells transfected with CPEC constructs of either WNV_KUN_ or PaRV with a CMV promoter. Monolayers were fixed 72 hrs post-infection. IFA analysis was performed by probing with anti-PaRV (7D11) or anti-WNV E (3.91D) mouse monoclonal antibodies. The nucleus of each cell was stained with Hoechst 33342. Images were taken at ×40 magnification”.

