# Supplementary material for: Infectious DNAs derived from insect-specific flavivirus genomes enable identification of pre- and post-entry host restrictions in vertebrate cells
Source: Sci Rep. 2017 Jun 7;7:2940. doi: 10.1038/s41598-017-03120-1 (PMC5462777; doi:10.1038/s41598-017-03120-1)
Supplement: Supplementary file 1 — Supplementary Data [file 41598_2017_3120_MOESM1_ESM.pdf]

Infectious DNAs derived from insect-specific flavivirus genomes enable identification of pre- and post-entry host restrictions in vertebrate cells

Thisun B.H Piyasena[1#], Yin X. Setoh[1#], Jody Hobson-Peters[1#], Natalee D Newton[1], Helle Bielefeldt-Ohmann[1], Breeanna J McLean[1], Laura J. Vet[1], Alexander A. Khromykh[1*] and Roy A. Hall[1*]

[1]Australian Infectious Diseases Research Centre, School of Chemistry and Molecular Biosciences, The University of Queensland, St Lucia 4072, Queensland, Australia.

[#] These authors contributed equally

*Address correspondence to Roy A. Hall, roy.hall@uq.edu.au and Alexander A. Khromykh, alexander.khromykh@uq.edu.au

Supplementary Data

Supplementary Table S1. Sequence comparison between PaRV$_{CPEC}$ and PaRV$_{CPECM1}$ showing a 15 nucleotide change in PaRV$_{CPECM1}$.

Sequence Name	Sequence
PaRV$_{WT}$	ACCTTGGAA - - - AGAGCCTCACGTGCGGAAGAGAAGGGAA
PaRV$_{CPEC}$	ACCTTGGAA - - - AGAGCCTCACGTGCGGAAGAGAAGGGAA
PaRV$_{CPECM1}$	ACCTTGGAAAGACGCAGCAGAAGGGCGGAAGAGAAGGGAA

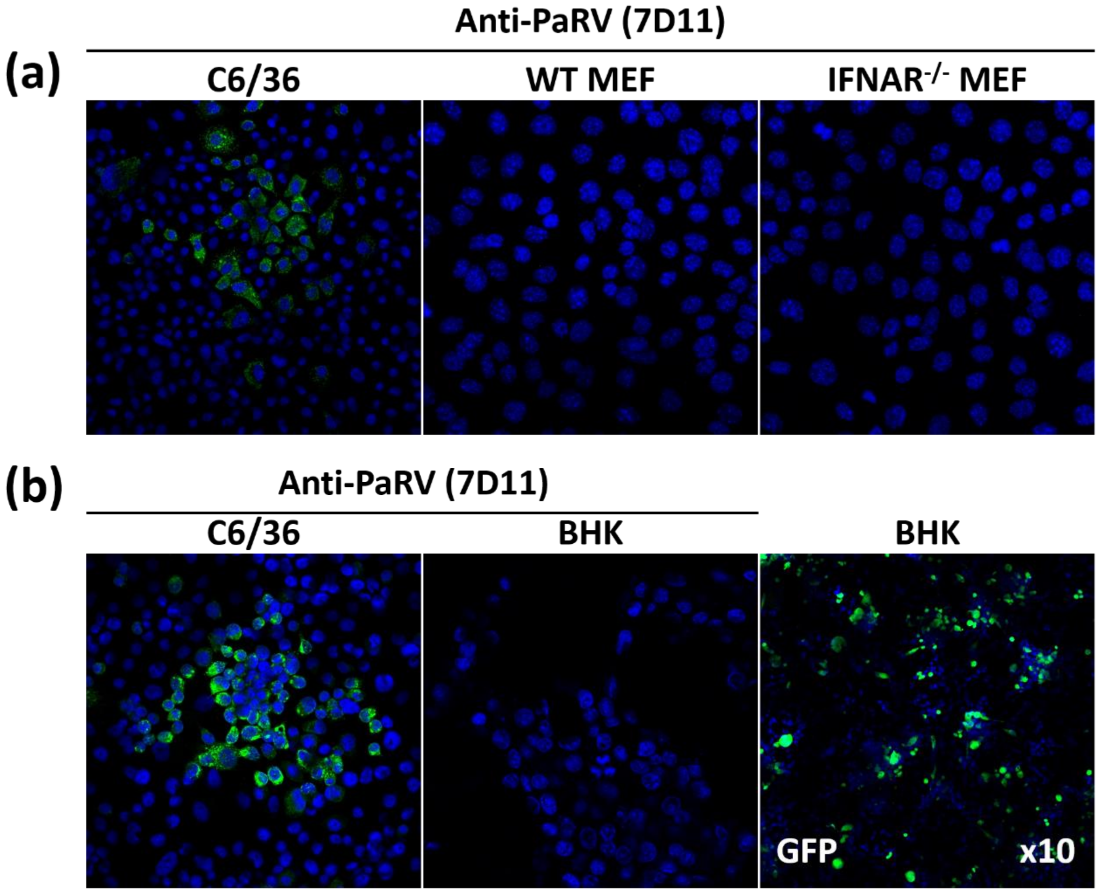

Supplementary Fig. S1. IFA visualisation of independent transfections of PaRV RNA into C6/36 cells and either (a) WT and IFNAR$^{-/-}$ MEF cells or (b) BHK cells. RNA from a GFP-nanoLuc fusion protein expressing WNV replicon was also included as a transfection control in BHK cells. IFA analysis was performed by probing with anti-PaRV E (7D11). Monolayers were fixed 72 hrs post-infection. IFA analysis was performed by probing with PaRV mouse anti-sera. The nucleus of each cell was stained with Hoechst 33342. Images were taken at ×40 magnification or as indicated.

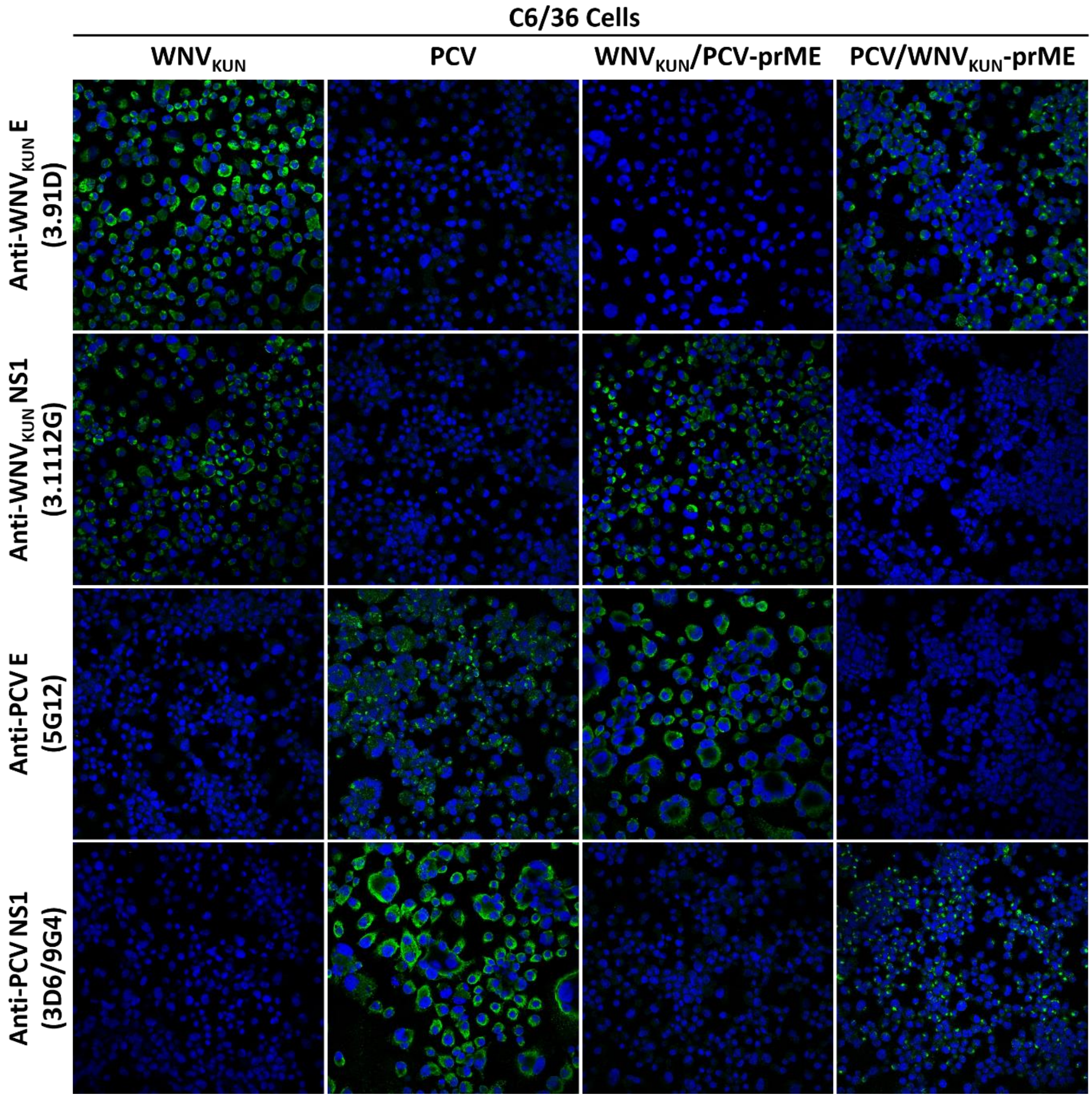

Supplementary Fig. S2. Visualisation of C6/36 cells infected with either WNV_{KUN}, PCV, PCV/WNV_{KUN}-prME or WNV_{KUN}/PCV-prME at an MOI of 1. Monolayers were fixed 72 hrs post-infection. IFA analysis was performed by probing with anti-PCV E (5G12), anti-PCV NS1 (3D6/9G4), anti-WNV E (3.91D) and anti-WNV NS1 (3.1112G) mouse monoclonal antibodies. The nucleus of each cell was stained with Hoechst 33342. Images were taken at ×40 magnification.
